# NADA Ear Acupuncture: An Adjunctive Therapy to Improve and Maintain Positive Outcomes in Substance Abuse Treatment

**DOI:** 10.3390/bs7020037

**Published:** 2017-06-16

**Authors:** Kenneth Carter, Michelle Olshan-Perlmutter, Jonathan Marx, Janet F. Martini, Simon B. Cairns

**Affiliations:** 1 Integrative Psychiatry Consultant, Keystone Substance Abuse Services, 199 S Herlong Ave, Rock Hill, SC 29732, USA; kocarter4@gmail.com; 2 Outpatient Psychiatry, Carolinas healthcare System BH, Charlotte 501 Billingsley Road, Charlotte, NC 28211, USA; 3 Professor of Sociology, Winthrop University, 334 Kinard Hall, Rock Hill, SC 29730, USA; marxj@winthrop.edu; 4 NCC Executive Director Keystone Substance Abuse Services, PO Box4437/199 S. Herlong Avenue, Rock Hill, SC 29732, USA; JMartini@keystoneyork.org; 5 Acupuncture Solutions, 220 Freeman Farm Road, Duncan, SC 29334, USA; Simon.cairns@att.net

**Keywords:** The National Acupuncture Detoxification Association protocol (NADA), ear acupuncture, substance abuse, behavioral health, addiction, acudetox

## Abstract

The National Acupuncture Detoxification Association protocol (NADA) is an adjunctive therapy using 1 to 5 invariant ear acupuncture/acupressure points. This is a randomized prospective study to determine if NADA plus traditional treatment enhance outcomes: quality of life, depression, anxiety and abstinence from substance abuse. There were 100 patients enrolled in the Keystone Substance Abuse Services-Winthrop University Department of Sociology and Anthropology NADA study. All patients completed Generalized Anxiety Disorder scale (GAD-7), Patient Health Questionnaire (PHQ-9), Quality of Life Enjoyment and Satisfaction Questionnaire (Q-LES) prior to starting the program and at program completion. Patients self-reported alcohol, tobacco, and drug use prior to starting the program at program completion and at 3 and 6 month follow- up. Patient characteristics are predictive of completion versus non-completion when race, criminal history and initial drug test is considered. Those identified as nonwhite, (*p* < 0.05) and patients with positive initial drug test, (*p* < 0.01) were more likely to complete treatment in the NADA group. Also, among patients with criminal history a higher percentage failed to complete the program in the control group (*p* < 0.05). Participation in NADA positively associated with Q-LES score (*p* < 0.05), feeling better about oneself and improved energy (*p* < 0.05), likelihood of employment upon discharge (*p* < 0.05), and decreased alcohol use at 3 month follow up (*p* < 0.05) and 6-month follow-up (*p* < 0.01). NADA group reported less tobacco use at 6 months (*p* < 0.05).

## 1. Introduction

The National Acupuncture Association protocol (NADA) is one of the most commonly used forms of acupuncture treatment in the United States. SAMSHA 2012 survey reported 628 licensed addiction treatment programs include acupuncture as a therapeutic tool [1]. NADA is a simple treatment that involves bilateral needle insertion at ear points Shen Men, Sympathetic, Kidney, Liver and Lung. Use of adjunctive NADA has been shown to relieve withdrawal symptoms and improve treatment engagement and retention [2,3]. Multiple studies published in peer reviewed journals support the adjunctive use of NADA for the treatment of heroin, alcohol, and cocaine addiction [4,5,6,7,8,9,10,11,12,13]. It can be used in the acute and chronic phases of substance use treatment. NADA is increasingly integrated into dual diagnosis settings to help patients with substance use disorders that also have other behavioral health symptoms. Patients reported significant benefits including improvement in depression, anxiety, anger, impaired concentration, and problems with energy and body aches/headaches [14]. NADA has shown to be effective in both successful completion of a 90 day inpatient dual-diagnosis treatment program for those with borderline personality disorder as well as tobacco cessation [15]. A systematic review and meta-analysis assessing the efficacy of auricular acupuncture examined 22 randomized clinical trials. Of these 13 were included for meta-analysis [16]. In these studies, ear acupuncture provided significant pain relief when compared to a sham or control group [16]. However, none of these studies specifically utilized NADA. A recent 2016 animal study using morphine dependent rodents showed that NADA intervention compared to 5 sham ear helix points prevented the development of morphine tolerance, reduced signs associated with cravings and shortened the time of analgesic effect of morphine [17]. Therefore, establishing an animal model of NADA can provide another tool for investigating the efficacy and cellular mechanisms of NADA treatment [17].

In addition, the NADA protocol is associated with a decrease in positive urine tests, increased program completion, improved patient satisfaction, and cost savings [18]. Total treatment cost is associated with lower medical costs and lower community psychiatric inpatient hospital costs [18]. Stahl examined the effect of mind body training, specifically, the Relaxation Response Resiliency Program (3RP) on healthcare utilization. Results of this retrospective study found mind body interventions such as 3RP reduced healthcare utilization at relatively low cost and thus may serve as a cost effective component in a health care delivery system [19]; NADA is also a mind body intervention that can be utilized in all aspects of a health care delivery system [20]. NADA induces a sense of “stillness” that is parallel to the relaxation response that patients experience with mind body based therapies [20]. In differentiating NADA from other mind body therapies it is clear that NADA is more passive in that participation can be entirely non-verbal and does not require a learning curve. NADA can be more effective than mind body based therapy in early abstinence and early stage recovery when individuals are likely to have a more limited capacity to actively participate in treatment, i.e., to be still, focus, concentrate and to learn and apply new information [20]. 

White conducted a systematic literature review on the use of acupuncture in addiction treatment [21]. Results were mixed regarding acupuncture benefit in abstinence, attrition, cravings, and withdrawal symptoms. There were notable disparities between patient reports of clinical benefit and the outcomes determined by researchers in clinical trials. Disparities may be understood to be due, in part, to the failure to capture statistical separation between two active treatments, i.e., the “active” and the so-called “sham” treatment. Studies using so-called “sham” acupuncture are less likely to be positive than those using non-acupuncture controls. Placing a needle any place on the body will elicit a response and thus “sham” acupuncture is not indeed a true sham that is an inactive control [21]. This suggests that the most reliable and clinically relevant NADA studies are those that compare a NADA group (NADA plus traditional treatment) to control group (traditional treatment alone). In fact, NADA is best understood as a somatic biopsychosocial intervention [22]. In a complicated holistic clinical setting, NADA research is best conducted using naturalistic or qualitative study design.

Keystone Substance Abuse Services serves as York County, South Carolina Act 301 substance abuse authority providing prevention, intervention, outpatient and inpatient services for all ages. Located in Rock Hill, South Carolina, (KSS) is situated on the border of the states North Carolina and South Carolina. It is the largest provider of treatment and prevention substance abuse services in York County, and is an integral care member of the Charlotte, North Carolina Greater-Metropolitan-Area healthcare network. KSS is a private, not-for-profit organization. In 2012 they adopted and implemented the Recovery Oriented System of Care treatment model. Patient assessment and treatment are congruent with best practice guidelines of the American Society of Addiction Medicine (ASAM). For more than a decade, KSS staff members have accumulated clinical experience using NADA protocol. Patients consistently reported liking and benefiting from NADA protocol included in the treatment program. KSS administrative and clinical leadership implemented this study in order to understand the ways in which NADA may be impacting treatment outcomes as an integrated component of the comprehensive services KSS provides. 

This study involves patients attending the KSS intensive outpatient program (IOP). KSS IOP utilizes ASAM criteria to determine program completion and length of stay. The program incorporates individual therapy, 12 Step orientation, and a variety of group therapies. Patient population at KSS is 90% referred from a variety of agencies such as probation and parole, Department of Social Services, Department of Motor Vehicle, the Recovering Professionals Program, employee assistance program, and physician offices. Ten percent are self-referred. KSS patients are high utilizers of a variety of healthcare services and have a high percentage of co-occurring psychiatric symptoms as determined on intake assessment. 

The objective of the study was to determine whether NADA plus traditional treatment is better than traditional treatment alone in improving and sustaining treatment outcomes including: quality of life and psychiatric symptoms, maintaining abstinence from drug, alcohol and tobacco, and decreasing healthcare utilization. The authors’ null hypothesis is that there is no significant difference in the outcomes between the group receiving NADA plus traditional treatment and the group receiving traditional treatment alone.

## 2. Materials and Methods

**Figure A1:**
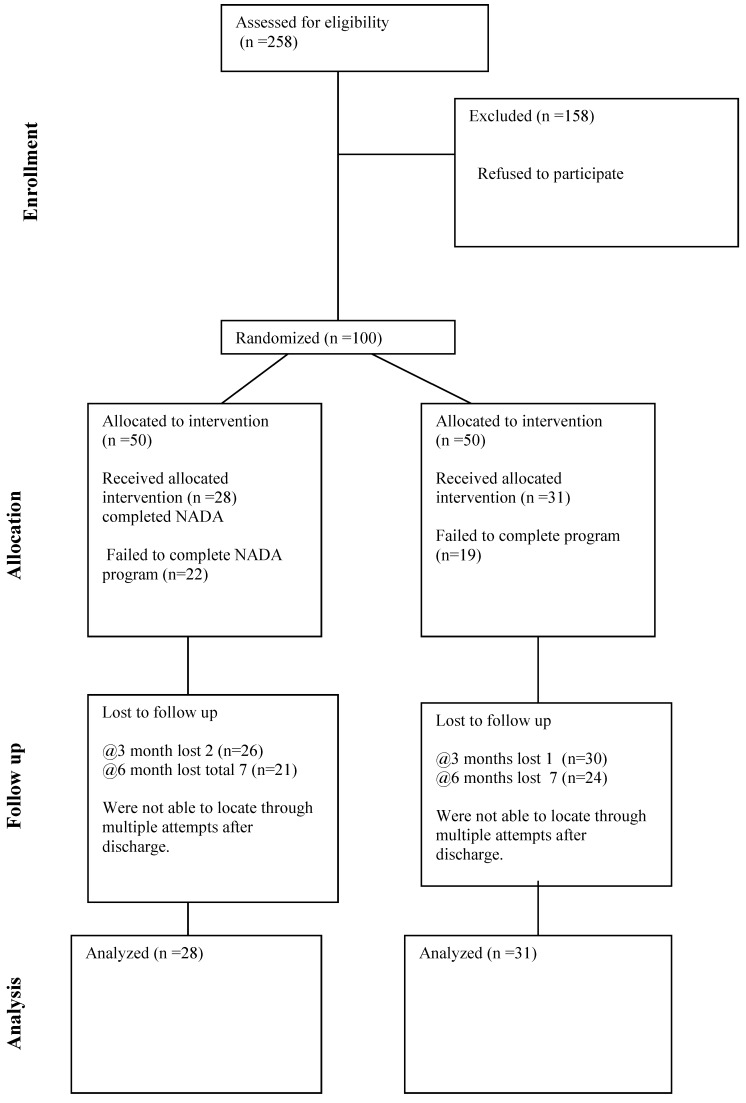
Flow chart.

A prospective randomized controlled trial (RCT) took place at KSS for patients entering the study between July 2015 and March 2016. During this time frame 258 people entered the KSS program. Each person was asked to participate; 158 declined and 100 were enrolled in the study (see flow chart in Figure A1). The sample size was chosen a priori based on general guidelines [23]. This study was a collaboration between KSS and the Winthrop University Department of Sociology and Anthropology. The study was approved by the Winthrop University Institutional Review Board (IRB). The study followed the National Institutes of Health ethical guidelines. Each patient signed informed consent prior to entering the study. Each qualified patient was given the choice of participating in this study which they understood included 3 and 6 month follow up assessment after program completion. The inclusion criteria included age 18–65 years old and a Diagnostic and Statistical Manual of Mental Disorders of the American Psychiatric Association Fifth Edition diagnosis of a substance use disorder. Patients could also have co-occurring mental health symptoms. Exclusionary criteria were as follows: (1) bruising, cut, or other lesions at the site of potential needle insertion; (2) unable or unwilling to follow directions for any reason; (3) younger than 18; (4) older than 65 years of age; (5) Non English speakers; and (6) involuntary commitment for alcohol and drug treatment. 

There were 100 patients enrolled in the study. NADA group (traditional treatment plus NADA), *n* = 50 or control group (traditional treatment only), *n* = 50. KSS staff utilized KSS electronic health records system and randomly assigned all new patients with a patient number, those that ended in an odd number were assigned NADA protocol group and those ending in an even number were assigned control group. 

Traditional treatment was provided to both NADA and control group. Traditional treatment included individual, 12 step orientation and a variety of group therapies. Both NADA and control groups were treated by the same group of therapists including certified substance abuse counselors and licensed clinicians. Patients in the control group participated in various didactic and process groups while patients in NADA group received NADA protocol. NADA group also participated in the same groups at other times during the day. Patients in NADA group received treatment twice weekly by NADA certified specialists/SC licensed Auricular Detoxification Therapists. Patients received NADA ear acupuncture in a group setting. Due to state law, NADA ear therapy may only take place under the direct on site supervision of a licensed acupuncturist or a person licensed to practice medicine under Article 6 Acupuncture Act of South Carolina. This did not affect the length of time for each NADA treatment i.e., (45 min per patient per group treatment session) but did profoundly affect the frequency at which we were able to make NADA ear treatment available. NADA ear treatment could only be offered on Tuesday and Wednesday of each week. The average length of stay was between 10–12 weeks for patients in both groups. The average number of NADA treatments was 10.14 (SD = 5.5) over an average of 8.3 weeks (SD = 2.9).

NADA group patients received twice weekly 5 bilateral ear acupuncture points while seated together in a large group of up to 20 patients per 45 min treatment sessions. The 5 specific ear points treated are Sympathetic, Shen Men, Lung, Kidney, and Liver points. FDA recognized needles (recognized: 21cfr88.5580) are inserted at the beginning of the treatment hour and generally remain in place for 30 to 45 min. Patients may request needle removal at any time. The acupuncture needles are sterile, single use, stainless steel shafts of 0.20 mm diameter and 7.0 mm length. The needles have bright fluorescent plastic handles. The acupuncture needles are provided in convenient sterile packages. Needles are inserted with a brief but steady movement. Ear needles penetrate approximately 1/8 inch, contacting the cartilage if it is present in that location. Needles are twirled 180 degrees for smoother insertion. 

Patients had to receive at least 2 acupuncture treatments to be included in the statistical analysis. Length of stay and program completion varied according to patients’ individual treatment plan which is guided by ASAM (American Society of Addiction Medicine) criteria and clinical judgment of the treatment team based on progress towards treatment goals, attendance, participation, and urine drug screens. A typical length of stay is four days a week for eight weeks. All patients completed Generalized Anxiety Disorder scale (GAD-7), Patient Health Questionnaire (PHQ-9), Quality of Life Enjoyment and Satisfaction Questionnaire (Q-LES) prior to starting the program and at time of discharge. In addition, patients self-reported alcohol, tobacco, and drug use, utilization of health care services at time of program completion and 3 and 6 months after program completion. 

The groups were compared at discharge on various dimensions: mean improvement on the Quality of Life Enjoyment and Satisfaction Questionnaire (Q-LES), Generalized Anxiety Disorder scale (GAD-7) and Patient Health Questionnaire (PHQ-9) depression scale. The groups were also compared using self-report at program completion, 3 months and 6 months after program completion for drug use, alcohol use, tobacco use and incidents of billable health care utilization.

Utilization of health care services was determined by asking each patient how many times they accessed outpatient/ER/inpatient services for physical care, mental health, and treatment for substance use in the last 30 days.

At 3 and 6 months after program completion there was a decline in the number of respondents. This was due to attrition secondary to inability to reach patients for follow up. There were 56 patients responding at 3 months and 45 patients responding at 6 months. There are slight variations based on non-response to particular items (see flow chart in Figure A1).

## 3. Results

Of the 100 patients enrolled 61 completed the KSS treatment program with no overall difference in completion rates between groups (NADA *n* = 28, 56%; Control *n* = 31, 62%; χ^2^ = 0.37, ns). (Table 1).

However, patient characteristics (see Table 1) completion/non-completion differ by race, criminal history and initial drug test categories. Among nonwhites a higher percentage complete the program in the NADA group (NADA *n* = 10, 36%, control *n* = 4, 13%; χ^2^ = 4.23 *p* < 0.05). Among patients with a positive initial drug test a higher percentage complete the program in the NADA group (NADA *n* = 21, 75%, Control *n* = 14, 42%; χ^2^ = 6, *p* < 0.01). Among those with criminal history a higher percentage failed to complete the program in the control group (NADA *n* = 8, 36%, Control *n* = 14, 73%, χ^2^ = 5.71 *p* < 0.05).

In Table 2, a mix measure analysis exploring psychological improvement of group by time illustrates a significant improvement for both treatment groups for anxiety/GAD-7 (NADA, χ^2^ = 12.22, *p* < 0.01; Control, χ^2^ = 6.48, *p* < 0.05) and depression/PHQ-9 ((NADA, χ^2^ = 13.45, *p* < 0.01; Control, χ^2^ = 8.09 *p* < 0.05). In a paired difference between means test (N = 17) indicates only those in the NADA group show a significant improvement in quality of life/Q-LES (Entry NADA $\bar{X}$ = 48; Discharge NADA $\bar{X}$ = 54.34, *t* = 3.04, *p* < 0.05). Post hoc tests reveal selected items in the PHQ (“feeling tired or having energy” and “feeling bad about yourself or that you let your family down”) showed statistically significant improvement for NADA group compared to the control group (The breakout data on these two items is available from the authors).

**Figure 1:**
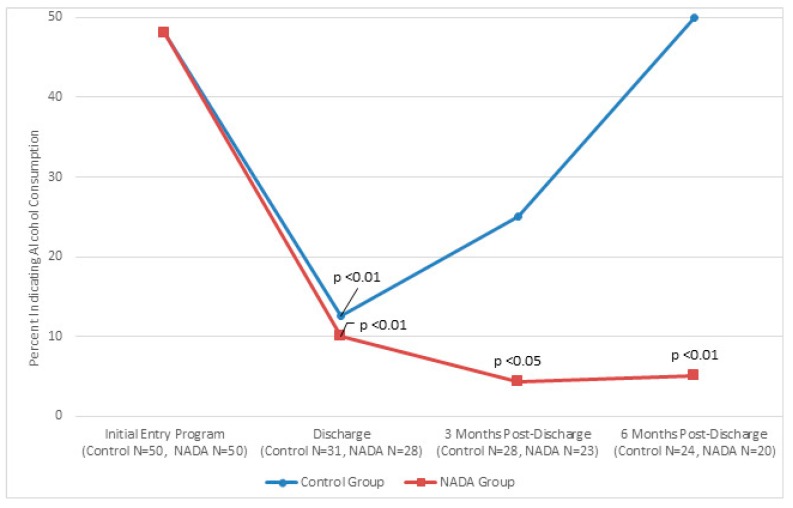
Percent of participants indicating alcohol use at initial entry into program, at discharge mark, 3 month post-discharge mark, and six month post-discharge mark for traditional and NADA treatment groups.

**Figure 2:**
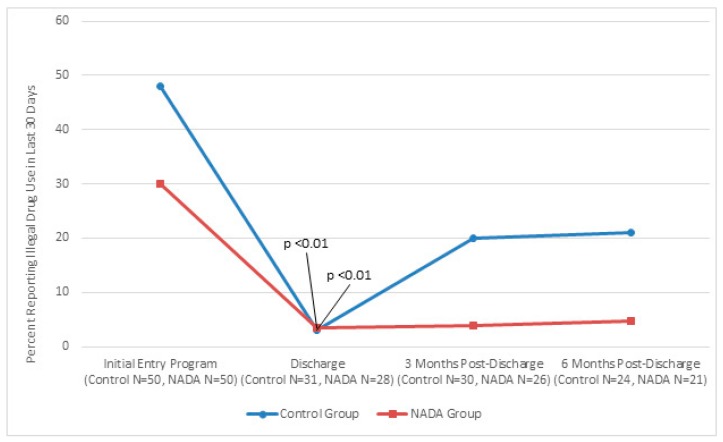
Percent of participants indicating illegal drug use at initial entry into program, at discharge mark, three months post-discharge mark, and six month post-discharge mark for traditional and NADA treatment groups.

**Figure 3:**
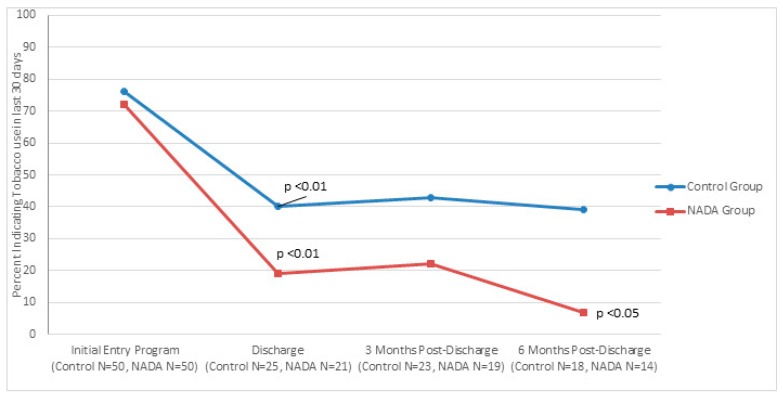
Percent of initial smokers completing the program that continued tobacco use at discharge mark, 3 month post-discharge mark, and six month post-discharge mark for traditional and NADA treatment groups.

Figure 1, Figure 2 and Figure 3 show traditional and NADA treatment improved patient’s maintaining abstinence from consumption in regard to alcohol (NADA, χ^2^ = 11.56, *p* < 0.01; Control, χ^2^ = 10.93, *p* < 0.01), drug (NADA, χ^2^ = 16.84, *p* < 0.01; Control, χ^2^ = 8.97, *p* < 0.01 and tobacco (NADA, χ^2^ = 16.58, *p* < 0.01; Control, χ^2^ = 10.37, *p* < 0.01) between program entry and program completion.

Consumption between groups significantly favored NADA at 3 months post discharge for alcohol (NADA *n* = 23, 4%; control *n* = 28, 25%, χ^2^ = 4.07, *p* < 0.05) and trended better for drugs and tobacco. This pattern continued at 6 months post discharge for alcohol consumption (NADA *n* = 20, 5%; control *n* = 24, 50%, χ^2^ = 10.61 *p* < 0.01). Tobacco consumption at 6 months significantly favored NADA (NADA *n* = 14, 7%, control *n* = 18, 39%, χ^2^ = 4.23, *p* < 0.05) and trended better for drug use compared to control group.

A reduction in healthcare utilization is found for both NADA and control group completers at time of discharge (NADA, χ^2^ = 20.14; Control, χ^2^ = 21.79; *p* < 0.01. Due to the small n each healthcare utilization category could not be separated out for analysis to draw meaningful inference. Healthcare utilization was grouped together and analysis is based on an indicator of general use versus nonuse. At 3 and 6 months the NADA group trended better than the control group in maintaining reduction in utilization of healthcare resources. 

With regard to employment, 50% of patients in NADA group and 54.8% patients in control group who completed the program were unemployed upon entering the program. At discharge 71% of patients in NADA group who previously were unemployed had attained some type of employment vs. 35% of control group (χ^2^ = 4.01, *p* < 0.05). 

## 4. Discussion

The main findings from this study support the hypothesis that NADA protocol when combined with traditional treatment improves QLES, feeling better about oneself and improved energy, likelihood of employment upon discharge, and decreased alcohol use at 3 month and 6 month follow up. NADA group also reported less tobacco use at 6 months.

Improvement in quality of life scores and symptoms associated with depression could be attributed to the physiological effects associated with acupuncture including changes in the production of neurotransmitters which influences the body’s regulator system and chemical balance [9]. This could also be influenced by the sense of stillness NADA provides similar to the relaxation response patients experience with mind body based therapies [20,24]. In differentiating NADA protocol from other mind body therapies, patient participation in NADA treatment can be passive in that participation can be entirely non-verbal. There is no delay in potential onset of efficacy due to the time required to actively learn about and to practice applying a mind body skill set. This fact can be especially important in early abstinence and early stage recovery when a patient’s performance is most likely to be impaired from substance use [20,24]. Thus, NADA treatment may advance the learning curve required for the psychosocial rehabilitation aspects of a comprehensive treatment program such as KSS by helping patients to be better able to learn skills required for successful recovery earlier in the recovery process.

The authors speculate improving patient’s sense of well-being helps improve outcomes in maintaining abstinence and improving likelihood of employment as well. Although NADA group and traditional group performed similarly with regard to anxiety and depression scores upon admission and discharge this schedule would not capture acute state effects associated with NADA treatment early on in the treatment program; a passive NADA treatment experience of a reduction in anxiety and depression whereby the patient “does nothing” may be associated with an accelerated sense of well-being reflected in observable behavior such as improved likelihood of employment, greater alcohol and tobacco abstinence. This may also be reflected in this study finding that patients in higher risk populations (nonwhite, criminal history, initial positive drug test) are more likely to complete the KSS treatment program when participating in NADA treatment.

The current trend in the literature suggests that NADA protocol when added to traditional treatment appears to be correlated with better outcomes in maintaining abstinence, improving overall health and sense of well-being. [14,15,25,26]. Chang et al. also reported patients receiving auricular acupuncture had greater improvement in the spirituality dimension of quality of life [26]. Lua et al. investigated auricular acupuncture for drug dependence using health related quality of life (HRQoL) as one of the measures. Even though results did not show any significant differences in either the methadone maintenance treatment (MMT) or the MMT+ auricular acupuncture (AA); the MMT+AA participants reported better HRQoL profiles compared to the MMT group, especially in overall HRQoL, general health, psychological domain [27]. Quality of life measures are just beginning to be included in studies using the NADA protocol as an adjunctive treatment. 

A growing body of literature supports our findings in maintaining abstinence from alcohol, drugs and tobacco with NADA protocol [2,4,10,13,15,25]. However, in reviewing the literature results have been mixed and many studies have been criticized due to the variations in methodological quality of the studies, high attrition at follow up, small sample size, and heterogeneity. In interpreting conflicting literature on the use of NADA protocol, [28,29] it is necessary to consider differences in psychosocial context and incentivization of trial participation. In the absence of clarity regarding treatment context it is difficult to draw definitive and accurate conclusions as to the clinical benefits from NADA protocol. 

The authors were hoping to shed some light on cost savings by looking at healthcare utilization. Reduction in healthcare utilization is found for both NADA and control group completers at time of discharge. Healthcare utilization categories were grouped together and analysis is based on an indicator of general use versus nonuse. At 3 and 6 months the NADA group trended better than the control group in maintaining reduction in utilization of healthcare resources. Due to the small sample size and inability to separate out categories for healthcare utilization it is difficult to ascertain cost benefit achieved by NADA group. Santasiero’s study utilizing the NADA protocol demonstrated cost savings were derived from higher program completion rates, increased number of negative urine screens, fewer inpatient rehabilitation days, fewer inpatient psychiatric days, and fewer outpatient detoxification episodes over the course of treatment [18].

### Study Limitations

The major limitation of our original project’s design is the lack of sample size estimation. Because existing empirical work on the topic is scarce, we did not have the required parameter estimates for the calculation. Furthermore, we confronted many logistical contingencies. We researched one center, and we could only gain access for a limited amount of time. The research team sought the largest logistically feasible sample to produce a powerful test (N = 100). It is our hope that our empirical work offers guidance to future researchers estimating sample sizes on related questions through the statistics/counts presented. It was difficult to obtain follow up information from patients after program completion. This resulted in a lower yield of patients available for follow up at 3 and 6 months. This was due to inability to reach many patients via telephone and due to the absence of a NADA specific aftercare component to attract and engage patients in follow up at the KSS treatment program location. 

In hindsight the absence of serial data regarding state effects of NADA protocol treatment is a variable that should be included in future studies in order to better understand the rate of improvement in target symptoms in the NADA protocol participants versus traditional controls.

This study was also impacted by the restrictive South Carolina law which requires direct on site supervision by either a physician or full body acupuncturist in order for NADA providers (referred to as Auricular Detoxification Therapists in South Carolina) to provide treatment. This creates a financial hardship for KSS program and for many other programs throughout the country where state laws require direct on site supervision. It would have been more affordable and easier to obtain a larger sample size in an environment that only required general supervision for NADA clinical staff. Where only general supervision is required NADA treatment can be provided with greater frequency, inexpensively and safely utilizing front line clinical staff (substance abuse counselors, nurses, social workers etc.).

## 5. Conclusions

Bearing in mind the study limitations discussed above, study findings suggest NADA protocol when combined with traditional treatment improves QLES, feeling better about oneself and improved energy and likelihood of employment upon discharge. It also suggests patients in higher risk populations (nonwhite, criminal history, initial positive drug test) are more likely to complete the KSS treatment program when participating in NADA treatment. Additionally, it helps to maintain abstinence from alcohol at 3 and 6 months follow up as well as tobacco at 6 month follow up.

Positive outcomes evidenced in this investigation need to be studied further and with larger sample sizes in order to better delineate NADA protocol impact on substance use and co-occurring mental health issues as well as the potential for cost savings.

## Figures and Tables

**Table 1:** Patient Characteristics.

	Total Patients (N = 100)	Completers (N = 59)			Non-Completers (N = 41)		
		NADA Group (N = 28)	Traditional Group (N = 31)	*p* Value ^1^	NADA Group (N = 22)	Traditional Group (N = 19)	*p* Value ^2^
Age							
Years ± SD	36.18 ± 12.7	36.44 ± 12.15	37.90 ± 12.56	0.66	35.54 ± 13.64	33.6 ± 13.6	0.66
Range	18–59	20–58	19–58	NS	18–58	18–59	NS
Race							
White	77 (78%)	18 (64%)	27 (87%)	0.04	19 (90%)	13 (68%)	0.08
Other	22 (22%)	10 (36%)	4 (13%)		2 (10%)	6 (32%)	NS
Gender							
Male	42 (42%)	10 (36%)	15 (48%)	0.43	9 (41%)	8 (42%)	0.94
Female	58 (58%)	18 (64%)	16 (53%)	NS	13 (59%)	11 (58%)	NS
Employment							
Employed	59 (59%)	14 (50%)	14 (45%)	0.71	9 (41%)	7 (37%)	0.79
Unemployed	41 (41%)	14 (50%)	17 (55%)	NS	13 (59%)	12 (63%)	NS
Criminal History							
Yes	47 (47%)	12 (44%)	13 (42%)	0.85	8 (36%)	14 (73%)	0.02
No	52 (53%)	15 (56%)	18 (58%)	NS	14 (64%)	5 (26%)	
Mental Health History							
Yes	59 (50%)	16 (57%)	16 (53%)	0.77	9 (41%)	8 (42%)	0.94
No	50 (50%)	12 (43%)	14 (47%)	NS	13 (59%)	11 (58%)	NS
Initial Drug Test							
Positive	62 (62%)	21 (75%)	14 (42%)	0.01	14 (64%)	14 (74%)	0.49
Negative	38 (38%)	7 (25%)	18 (58%)		8 (36%)	5 (26%)	NS

^1^ *p* value for NADA Group versus Traditional Group within completers. ^2^ *p* values for NADA Group versus Traditional Group within Non-completers.

**Table 2:** Psychological Improvement of Completers by Treatment Group Over time.

	All Completer (N = 57)			Traditional Group			NADA Group		
	Entry	Discharge		Entry	Discharge		Entry	Discharge	
GAD7									Anxiety
Severe >15	26 (46%)	3 (9%)	*p* < 0.000	14 (45%)	2 (14%)	*p* < 0.05	12 (46%)	1 (5%)	*p* < 0.01
Moderate 6–14	23 (40%)	14 (41%)		13 (42%)	6 (43%)		10 (39%)	8 (40%)	
Mild <5	8 (14%)	17 (50%)		4 (13%)	6 (43%)		4 (15%)	11 (55%)	
PHQ-9									Depression
Severe >15	21 (36%)	1 (3%)	*p* < 0.000	11 (35%)	1 (7%)	*p* < 0.05	10 (36%)	0 (0%)	*p* < 0.01
Moderate 6–14	29 (49%)	14 (41%)		16 (52%)	7 (47%)		13 (46%)	7 (37%)	
Mild <4	9 (15%)	19 (56%)		4 (13%)	7 (47%)		5 (18%)	12 (63%)	
Q-LES ~				Quality					Life
Mean	48	52	*p* < 0.21	47.9	48.8	*p* < 0.44	48	54.35	*p* < 0.05
			NS			NS			

~ Paired Difference between Means.

## Supplementary Materials

The following are available online at www.mdpi.com/2076-328X/7/2/37/s1.
