# Supplementary material for: NADA Ear Acupuncture: An Adjunctive Therapy to Improve and Maintain Positive Outcomes in Substance Abuse Treatment
Source: Behav Sci (Basel). 2017 Jun 16;7(2):37. doi: 10.3390/bs7020037 (PMC5485467; doi:10.3390/bs7020037)
Supplement: Supplementary file 1 [file behavsci-07-00037-s001.docx]

| **PMC full text:** | [BMJ. 2010; 340: c332.](https://www.ncbi.nlm.nih.gov/pmc/articles/PMC2844940/)  Published online 2010 Mar 23. doi:  [10.1136/bmj.c332](https://dx.doi.org/10.1136%2Fbmj.c332)  [Copyright/License ►](https://www.ncbi.nlm.nih.gov/pmc/articles/PMC2844940/table/tbl1/)[Request permission to reuse](https://www.ncbi.nlm.nih.gov/pmc/about/copyright/) |  |
| --- | --- | --- |

CONSORT 2010 checklist of information to include when reporting a randomised trial*

| **Section/Topic** | **Item No** | **Checklist item** | **Reported on page No** |
| --- | --- | --- | --- |
| **Title and abstract** | | | |
|  | 1a | Identification as a randomised trial in the title | Line 16 |
|  | 1b | Structured summary of trial design, methods, results, and conclusions (for specific guidance see CONSORT for abstracts^21 31^) | Line 15-31 |
| **Introduction** | | | |
| Background and objectives | 2a | Scientific background and explanation of rationale | Line 40-89 |
|  | 2b | Specific objectives or hypotheses | Line 111-115 |
| **Methods** | | | |
| Trial design | 3a | Description of trial design (such as parallel, factorial) including allocation ratio | Line 134-188 |
|  | 3b | Important changes to methods after trial commencement (such as eligibility criteria), with reasons | N/A |
| Participants | 4a | Eligibility criteria for participants | Line 126-132 |
|  | 4b | Settings and locations where the data were collected | Line 104=111 and Line 111 |
| Interventions | 5 | The interventions for each group with sufficient details to allow replication, including how and when they were actually administered | Line 140-165 |
| Outcomes | 6a | Completely defined pre-specified primary and secondary outcome measures, including how and when they were assessed | Line 172-176 and Line 178-189 |
|  | 6b | Any changes to trial outcomes after the trial commenced, with reasons | No changes |
| Sample size | 7a | How sample size was determined | One limitation of our original projects design is the lack of sample size estimation. Because existing empirical work on the topic is scarce, we did not have the required parameter estimates for the calculation. Furthermore, we confronted many logistical contingencies.  We researched one center, and we could only gain access for a limited amount of time.  The research team sought the largest logistically feasible sample to produce a powerful test (N=100).  A   Larger sample size would have been advantageous. It is our hope that our empirical work offers guidance to future researchers estimating sample sizes on related questions through the statistics/counts presented. |
|  | 7b | When applicable, explanation of any interim analyses and stopping guidelines | N/A |
| Randomisation: |  |  | Line 137-139 |
| Sequence generation | 8a | Method used to generate the random allocation sequence | Every other number |
|  | 8b | Type of randomisation; details of any restriction (such as blocking and block size) | N/A |
| Allocation concealment mechanism | 9 | Mechanism used to implement the random allocation sequence (such as sequentially numbered containers), describing any steps taken to conceal the sequence until interventions were assigned | N/A |
| Implementation | 10 | Who generated the random allocation sequence, who enrolled participants, and who assigned participants to interventions | Line 137-139 |
| Blinding | 11a | If done, who was blinded after assignment to interventions (for example, participants, care providers, those assessing outcomes) and how | N/A |
|  | 11b | If relevant, description of the similarity of interventions | N/A |
| Statistical methods | 12a | Statistical methods used to compare groups for primary and secondary outcomes | Line 235-242 |
|  | 12b | Methods for additional analyses, such as subgroup analyses and adjusted analyses | Line 235-242 |
| **Results** | | | |
| Participant flow (a diagram is strongly recommended) | 13a | For each group, the numbers of participants who were randomly assigned, received intended treatment, and were analysed for the primary outcome | See Flow chart |
|  | 13b | For each group, losses and exclusions after randomisation, together with reasons | See Flow chart |
| Recruitment | 14a | Dates defining the periods of recruitment and follow-up | Line 119-120 |
|  | 14b | Why the trial ended or was stopped | Participant enrollment completed to the extent possible with the limited resources available |
| Baseline data | 15 | A table showing baseline demographic and clinical characteristics for each group | See demographic table |
| Numbers analysed | 16 | For each group, number of participants (denominator) included in each analysis and whether the analysis was by original assigned groups | Line 203-295 |
| Outcomes and estimation | 17a | For each primary and secondary outcome, results for each group, and the estimated effect size and its precision (such as 95% confidence interval) | Alpha =.05 |
|  | 17b | For binary outcomes, presentation of both absolute and relative effect sizes is recommended | N/A |
| Ancillary analyses | 18 | Results of any other analyses performed, including subgroup analyses and adjusted analyses, distinguishing pre-specified from exploratory | N/A |
| Harms | 19 | All important harms or unintended effects in each group (for specific guidance see CONSORT for harms^28^) | There were no adverse events. No one withdrew from the trial due to harms. Therefore, there was no harms data collected |
| **Discussion** | | | |
| Limitations | 20 | Trial limitations, addressing sources of potential bias, imprecision, and, if relevant, multiplicity of analyses | Line 388-410 |
| Generalisability | 21 | Generalisability (external validity, applicability) of the trial findings | Line 307-356 |
| Interpretation | 22 | Interpretation consistent with results, balancing benefits and harms, and considering other relevant evidence | Line 307-356 |
| **Other information** | | |  |
| Registration | 23 | Registration number and name of trial registry | IRB approval number IRB 15101  Federal Wide Assurance number FW A00012546 and IRB-IORG Registration Number IORG0005181   \|  \| \| --- \| |
| Protocol | 24 | Where the full trial protocol can be accessed, if available | Sponsored Programs & Research (SPAR)Winthrop University149 McLaurin Hall Rock Hill, SC 29733, USA[803/323-2460](tel:(803)%20323-2460) [803/323-4893](tel:(803)%20323-4893) (fax) |
| Funding | 25 | Sources of funding and other support (such as supply of drugs), role of funders | No Funding |
